# Voting with their feet: Primary care provider choice and its implications for public sector primary care services in India

**DOI:** 10.1016/j.socscimed.2023.116414

**Published:** 2024-01

**Authors:** Krishna D. Rao, Akriti Mehta, Caitlin Noonan, Michael A. Peters, Henry Perry

**Affiliations:** Department of International Health, Johns Hopkins University, Baltimore, MD, USA

**Keywords:** Primary health care, Bypassing, India, Informal providers, Provider choice, Health systems

## Abstract

Expanding networks of government primary health centers (PHCs) to bring health services closer to communities is a longstanding policy objective in LMICs. In pluralistic health systems, where public and private providers compete for patients, PHCs are often not the preferred source for care. This study analyzes the market for primary care services in the Indian state of Bihar to understand how choice of primary care provider is influenced by distance, cost and quality of care. This study is based on linked surveys of rural households, PHCs, and private primary care providers conducted in 2019 and 2020. Most rural residents lived in proximity to a primary care provider, though not a qualified one. Within a 5-km distance, 60% of villages had a PHC, 90% had an informal provider, 35% an Indian systems of medicine practitioner, and 10% a private MBBS doctor. Most patients sought care from informal providers irrespective of PHC distance; only 25% of patients living in the PHC's vicinity sought care there. Reducing distance to the PHC by 1 km marginally increased the likehood of the PHC being selected, and reduced the likelihood of private clinics being selected. Reducing patient's costs at PHCs increased the likelihood of the PHC being selected and reduced the likelihood of private clinics and private hospitals being selected. Improved clinical quality at PHCs had no effect on patient selection of PHCs, private clinics, or hospitals. Illness severity reduced the likelihood of PHCs or private clinics being selected, and increased the likelihood of private hospitals selected. Wealthier patients were marginally more likely to use PHCs, substantially more likely to use private hospitals, and less likely to use private clinics. Expanding PHC network coverage or improving their quality of care is not sufficient to make PHCs more relevant to local health needs. An orientation towards essential public health functions, as well as, a community-centered approach to the organization of primary health care system is necessary.

## Highlights

• Most patients bypassed local primary health centers for informal providers.
• The likelihood of a patient visiting any health facility declined with distance.
• Illness severity or wealth had no effect on choice of private clinics over PHCs.
• Illness severity or wealth increased preference for private hospitals over PHCs.
• Making health centers relevant requires attention to felt needs of communities.

## 1 Introduction

The aspiration to bring ‘health care as close as possible to where people live and work’ has been a longstanding aim of primary health care policy in many low- and middle-income countries (LMICs) (International, 1978). Beginning in the post-World War 2 period, countries made substantial investments in expanding networks of rural health facilities that offered promotive, preventive, and curative health services to catchment populations (Berman, 2000). The limitations of health services organized around fixed service delivery points became apparent by the early 1970s with the recognition that government health facilities, even when augmented with extension services, were neither able to reach everyone nor to fully cater to local health needs (Berman, 2000). This period also witnessed the expansion of community health worker programs which provided another approach to bringing health services closer to people using lay health workers (Berman, 2000; Perry et al., 2014). Nevertheless, rural health centers continue to occupy a central role in government policy. As an example, India's new primary health care strategy, the Ayushman Bharat-Health and Wellness Center Program (HWC), aims to establish 150,000 ‘Health and Wellness Centers’, which are upgraded primary health care facilities, close to rural communities (Government of India, 2018). These HWCs are expected to become “the first port of call for each family to access a full range of primary care services” (GOI. Ayushman Bharat Health, 2022).

Publicly financed personal health services delivered through health centers can have large impacts on population health and financial protection (Berman, 2000). Preventive services such as immunizations, as well as curative services, address health conditions that comprise a large share of national disease burden (Berman, 2000). The majority of Disability-adjusted life years (DALYs) lost globally and the top causes of disease burden in children under five years of age are addressed primarily through personal health services provided in outpatient settings (Berman, 2000). Estimates based on the Disease Control Priorities-3 suggest that if evidence-based interventions offered in communities (including health posts) and at health centers reach all those in need, 77% of maternal, newborn, and child deaths and stillbirths could be avoided (Black et al., 2016). Further, most health services required to manage cardiovascular, respiratory, or related disorders (CVRD), which are responsible for the majority of adult deaths, can be delivered through outpatient services at health centers (Prabhakaran et al., 2017). As such, functional outpatient health centers can contribute to large improvements in population health (Berman, 2000).

Expenditures on outpatient curative services comprise a large share of out-of-pocket (OOP) payments. In LMICs, where financial protection systems have limited coverage, expenditure on personal curative services is a leading contributor to catastrophic health expenditures and related impoverishment. As an example, in Kenya, outpatient care was responsible for 8% of the population experiencing catastrophic health expenditures compared to 2% from inpatient care; in India, eliminating OOP payments for outpatient care will reduce the percentage of households experiencing catastrophic health expenditures from 5% to 0.8% (Salari et al., 2018; Shahrawat and Rao, 2012). Because privately provided outpatient care is costlier to patients relative to care in publicly financed health facilities, the latter can potentially reduce financial hardship in catchment populations.

In pluralistic health systems, health care markets are characterized by public and private providers competing for patients (Meessen et al., 2011). This is particularly true of markets for primary care (curative) services; in many LMICs, the public system is not the main provider of primary care. This reduces the agency of government health centers to influence population health. As an example, a survey of 70 LMICs showed that overall, more than half the children ill with diarrhea, fever, and cough were treated by non-state (private) providers, though public sources were the main providers for child birth (38% by private providers) and preventive services (Grépin, 2016). In a sub-sample of 38 countries, nearly half had more than 40% of diarrhea or fever and cough cases treated by private providers. Expectedly, the population covered by networks of government health facilities is an important determinant of the extent to which they cater to population health needs; several studies have reported that utilization of government health centers is inversely related to the distance between the center and patients’ residence (Gabrysch et al.; Yao and Agadjanian, 2018; Nesbitt et al., 2016; Oldenburg et al., 2021). However, patients often bypass the local government health center, even when living in its vicinity, to seek services elsewhere, often at greater cost (Yao and Agadjanian, 2018; Akin and Hutchinson, 1999; Kahabuka et al., 2011; Kant et al., 2016; Khan et al., 2014; Rao and Sheffel, 2018). Such care seeking dynamics are more prevalent in pluralistic health care markets.

This study draws attention to how conventional notions of the significance of government health centers to the curative healthcare needs of local communities can be rather simplistic in the context of pluralistic health systems. Using the state of Bihar in India as an example, this paper aims to identify the types of first-contact primary care providers serving rural communities; second, understand how choice of primary care provider is influenced by provider attributes and patient characteristics; and third, discuss implications for primary health care policy. Note that the paper focuses on treatment of acute illnesses and injuries, and not on other health services like immunizations, wellness care, deliveries, chronic disease management, or family planning.

### 1.1 Study context

The study is located in the state of Bihar in eastern India. The state is among the economically poorest in India with health indicators well below the national average. For example, infant mortality rate in Bihar (India) is the second highest in the country at 46.8 (36.2) deaths per 1000 live births (International Institute for Population Sciences (IIPS) and ICF, 2021). It also has the lowest density (1.5 per 10,000) of qualified health workers, doctors (0.3 per 10,000) and nurses & mid-wives (0.4 per 10,000) in India (Rao et al., 2016a). For comparison, India has an average density of 9.1 qualified health workers, 3.4 doctors and 3.2 nurses and mid-wives per 10,000 population (Rao et al., 2016b). In this context, the need for government provided health services is anticipated to be high. Like elsewhere in India, Bihar's primary care markets are highly pluralistic with a mix of public and private qualified clinicians practicing allopathic and Indian systems of medicine (AYUSH^1^), as well as a large number of informal providers with no formal training in medicine.

## 2 Methods

This study is based on surveys of rural households and primary care providers conducted in the state of Bihar between November 2019 and March 2020. For a sub-sample of the household survey, ill household members were linked with first contact primary care providers from whom they sought services. Details of the surveys are provided below:

Household survey: Information was collected from rural households on different aspects of their health and socioeconomic condition. Of particular relevance, the survey recorded household reports of illness and care seeking – sickness or injury in the past one month, hospitalizations in the past year, vaccination in children less than two years of age, and births in the past year. Details of where household members sought care, number of providers visited, and how much they spent OOP on health care was collected. In addition, information on household asset ownership was recorded. Individual family members or a competent adult responded to the survey questions.

The household sample was selected as follows: From 9 divisions of Bihar, 70 administrative blocks (out of 534) were selected by stratified random sampling; the number of blocks selected in each division was proportionate to the relative size of division's blocks to the total blocks. Each block has one Block Primary Health Center (PHC). In each block, five villages were selected by probability proportional to size (PPS) sampling based on village population. Within each village 30 households were selected using the random walk technique i.e. from the center of the village a random direction was selected and every alternate household in that direction was sampled until the quota of 30 households was reached. Within the overall target sample size of 10,500 households, a total of 8,356 households (80% response rate) across 343 villages in Bihar were sampled comprising 39,477 individuals.

Block PHC survey: All 70 of the Block PHCs associated with the selected blocks were surveyed. In 66 of the 70 PHCs, we were able to observe structural and clinical quality of the main clinician present (usually the Medical Officer). Blocks vary in population size from less than 100,000 to around 350,000. The Block PHC is the main government health center in the block and has associated Additional PHCs and health sub-Centers, though these facilities have variable functionality. A Block PHC should be staffed with specialist and general doctors, however typically only general (MBBS) and/or AYUSH doctors are present. Other key staff include nurses, pharmacists, lab technicians, and auxiliary nurse-midwifes. Block PHCs can have between 6 and 30 beds. They offer preventive and curative outpatient care, as well as family planning and childbirth services. In a surveyed Block PHC, the main clinician's knowledge in treating common illnesses was assessed using clinical vignettes for hypertension in adults, and diarrhea and pneumonia in children. Further, an assessment of equipment and drug availability, and building conditions was made to evaluate structural quality.

Interlinked household and first contact primary care provider survey: From the five surveyed villages per block in the household survey, one village was randomly selected wherein primary care providers located within 5 km of the village were identified and interviewed. In these 70 villages comprising 1,744 sampled households, an attempt was made to identify and interview providers who had treated household members in the past month. In 57 of these 70 villages, we were able to achieve a reasonable mapping of the local health care market; in each of these 57 villages, we were able to link at least 40% of patient visits to a provider who was interviewed (in 56 of these villages, at least half the patient visits were linked to a provider). In these 57 villages together, 1909 individuals sought treatment outside home in the month before the survey, and we linked 1151 (60%) visits to a provider yielding a total of 262 unique primary care providers. We excluded patients who visited drug shops.

### 2.1 Key variables and analytics

One of our main variables of interest is distance of households from primary care providers. Latitudes and longitudes of households and primary care providers were collected in the World Geodic System (WGS84) datum. We used Stata's ‘geodist’ command to compute geodetic distances using the Vincenty formula, which calculates the length of the shortest curve between two points along the surface of a mathematical model of the earth (Vincenty, 1975). Distances were computed between households, the providers they visited, and the local PHC.

Primary care providers: There are two broad types of health centers surveyed – government PHCs and private clinics. We excluded drug shops and pharmacies because no formal consultations are conducted there. The main clinician present at the PHC was interviewed – these were mostly MBBS doctors, and some AYUSH physicians. Private providers were identified by where patients sought care for acute illnesses and were classified based on their qualification self-reports into the following categories: MBBS doctor (includes post-graduate training in medicine), AYUSH physician, and informal providers (IP) – includes individuals with training in pharmacy, and individuals possessing no formal medical training.

Provider competence: Public and private sector provider competence was assessed using clinical vignettes whereby the interviewer acted as the patient and a simulated consultation was carried out with the provider (Rao and Sheffel, 2018; Leonard and Masatu, 2005; Das and Hammer, 2005; Rao et al., 2013). Providers were assessed on management of three standardized cases - diarrhea with severe dehydration in an infant boy, pneumonia in a young girl, and hypertension in a middle-aged woman. These conditions have a high disease burden in Bihar. The core areas for assessment in each vignette included history, examination, diagnostics, diagnosis, prescription, and home care recommendations. The items in each core area of assessment represent quality actions that a clinician should do and were selected in a multi-stage process based on standard treatment guidelines, consultations with primary care practitioners in Bihar, and experts. The competence scores derived from the clinical vignettes measure how much providers know about managing the presented condition. For each clinical care provider, items were summed for each section, and section scores were summed across cases and scaled so that the competence score ranged from 0 to 100.

Household economic status: At the household level, a wealth index was constructed based on assets available in the household using principal component analysis (Filmer and Pritchett, 2001). Individuals were assigned to one of five quintiles based on the household wealth index.

Modelling provider choice: When someone falls ill in rural Bihar and decides to seek medical advice, they can potentially seek care from several types of providers – public sources such as community health workers, the Block PHC (including lower level facilities), and district hospitals; or, private sources such as – clinics of private doctors and AYUSH practitioners, private hospitals, informal practitioners, and drug shops/pharmacies. In the household survey, patients reported where they sought outpatient care as – community health worker, government clinic, government hospital, private doctor/clinic, private hospital, traditional healer, pharmacy/compounder, or other. In our analysis, we combined all public sources into the category of PHC; government community health workers are affiliated with PHCs and only a small proportion of patients sought care at district hospitals. Private clinics (including AYUSH/traditional practitioners) and private hospitals were retained as separate categories, and we did not include pharmacy/compounder in the analysis. The vast majority of private providers are informal providers, as shown in our and other studies (Rao et al., 2016a; Gautham et al., 2011, 2014).

Once patients have made a decision to seek medical advice outside their home, their choice of provider depends on factors related to attributes of the provider (e.g. distance, quality, cost), as well as, patient characteristics (e.g. severity of illness, age, sex, economic status). Our interest is in understanding the effect of provider attributes and patient characteristics on provider choice once a decision to seek care has been made. According to random utility theory, individual (i) selects provider (j), from the available set of providers, that maximizes their utility ($U_{\text{ij}})$ (Wiseman et al., 2008). $U_{ij}=\beta X_{ij}+\varepsilon_{ij}$, where $\beta X_{\text{ij}}$ is a vector of observable provider attributes and patient characteristics and their coefficients. The probability of choosing provider alternative j is a function of the selected provider attributes and of other providers in the patient's community, as well as patient characteristics: $\mathrm{Pr}\left(j=1\right)=\frac{\exp\left(\beta X_{ij}\right)}{\Sigma_{3}\left(\beta X_{ij}\right)}$.

We first fit a multinomial logit regression model and then a random parameters logistic regression model (cmmixlogit in Stata) to estimate the probability of a respondent selecting provider j. In this model, as discussed above, individuals choose between three alternative providers – PHC, private clinic (mostly informal providers), and private hospitals. The random parameters regression model incorporates correlation interdependence between alternatives through the random parameters; this relaxes the independence of irrelevant alternatives (IIA) assumption that the alternatives are uncorrelated, so that the inclusion/exclusion of an alternative will not alter the probabilities of choosing remaining alternatives (Wiseman et al., 2008; Mulcahy et al., 2021; Sarkodie, 2022). The presented results are based on the random parameters logistic regression model.

The main independent variables in the model include, provider distance from patients’ home, provider clinical quality of care, and patient cost of visit. Distance was based on patient reports of distance for private facilities and geodetic distance for PHCs. The cost of the visit was estimated as OOP payments made by the patient for consultation, drugs, and diagnostics. Clinical quality was assessed using clinical vignettes. Other independent variables relate to patient characteristics: age, sex, severity of illness, and economic status measured as the household wealth index quintile. These variables are described in Table 1.

**Table 1:** Sample characteristics.

	All	Study sample^a^
**Household Survey: Patient characteristics**		
N (patients)	10,617	7279
First contact provider visited (%)		
Primary Health Center	3.8	6.1^b^
Private clinic (incl traditional healers)	68.2	87.0
Private hospital	5.9	6.6
Government hospital	1.3	–
Pharmacy/drug shop	20.8	-c
Out-of-pocket health expenditure (Rs.)		
Primary Health Center	315.8 (235.4, 396.1)	284.2 (202.9, 365.6)
Private clinic (incl traditional healers)	601.8 (570.5, 633.0)	572.2 (546.2, 598.2)
Private hospital	3056.2 (2557.0, 3555.4)	2201.9 (1960.5, 2443.4)
Male (%)	44	44
Distance to provider (Km)	4.6 (4.4, 4.7)	5.1 (5.0, 5.2)
Age	30.6 (30.2, 31.0)	30.0 (29.5, 30.5)
Asset wealth quintile		
Poorest 20%	22	23
Quintile 2	27	28
Quintile 3	12	12
Quintile 4	20	19
Richest 20%	20	19
Severely ill (%)	19	20
**Interlinked Patient-Provider Survey**		
N (providers)	262	–
Provider type (%)		
Primary Health Center	11	–
Private MBBS doctor	3	–
Private AYUSH physician	12	–
Private Informal provider	74	–
Provider age (years)	44.4 (42.6, 46.2)	–
Male (%)	97	–
Provider competence		
PHC clinician	40.4 (36.7, 43.2)	40.1 (39.0, 41.1)
Private MBBS doctor (0–100)	31.4 (26.8, 36.0)	–
Private AYUSH practitioner (0–100)	30.7 (28.0, 33.4)	–
Private informal provider (0–100)	27.7 (26.4, 29.0)	
Private clinician	–	28.8^d^ (28.6, 28.9)

Note (International, 1978): Figures in parenthesis are 95% CI. (Berman, 2000).

a Study sample for regression analysis.

b Includes government hospitals.

c Dropped from analysis.

d Includes all private clinicians.

Because we only observe the attributes of the provider visited by the patient, to determine attributes of the alternatives not selected we made the following adjustments: for distance we used the patient-reported average distance to that provider type in that village or district (except for PHC distance, which was directly measured). For quality of care we used average score from the clinical vignettes for that provider type in the block or district where the patient was located. Clinical quality of providers was directly measured for PHCs, and for private hospitals we used the average vignette score of private MBBS doctors. For costs we used predicted values of the level of patient OOP expenditure by provider type, with illness severity, age, and household wealth quintile as covariates.

Endogeneity of patient costs: In provider choice models endogeneity can arise due to several reasons inducing a correlation between the regression error term and independent variables (Cavatorta et al., 2021; Petrin and Train, 2010). These include unobserved variables that affect provider choice (e.g. unobserved patient perceptions of quality correlated with prices), or when situations of reverse causality are created by, for example, providers increasing prices in reaction to increased demand for their services. In choice models related to demand for health services, concerns about endogeneity have focused on the endogeneity of prices due to likely correlation between prices and the error term (Cavatorta et al., 2021; Petrin and Train, 2010). We address this using the control function approach (Petrin and Train, 2010). In the control function method, a proxy variable is estimated that conditions on the part of patient costs that is correlated with the error term thereby allowing the regression error term to be independent of endogenous variable in the model. We implement the cost function approach by (a) identifying an exogenous instrumental variable that is related to patient costs but not the utility derived from the visit; (b) regress observed patient costs on the cost instrumental variable and patient characteristics (sex, age, illness severity, wealth quintile) and estimate the residuals; and (c) include the estimated residuals in the main regression as an additional control variable. Following Petrin and Train (2009) we estimate the cost instrument as the average cost of health care from a particular type of provider in markets other than the one where the patient is present. We consider a district as a health market. This is an appropriate instrument because the costs of the same type of provider in different markets reflect common costs but not common demand shocks (Cavatorta et al., 2021). Separate regressions were run for each type of provider. Regression results with and without using the cost function are shown in Table A1.

### 2.2 Ethics and permission

Ethical approval was granted by the Indian Institutional Review Board in India (Reference number: 10015/IRB/19–20) as well as the Johns Hopkins University Institutional Review Board. In the household survey, the purpose of the study was explained to each individual of the household and written informed consent was obtained from each respondent. If the respondent agreed to participate, then a signed copy of the consent form was provided to the respondent. For the provider survey, oral consent was obtained from the providers.

## 3 Results

In the household survey, of the 39,477 individuals surveyed, 10,617 individuals were sick in the past 30 days and had sought treatment outside home. Our study sample for the regression analysis is based on 7279 individuals who were not hospitalized for their illness, did not visit a drug shop for treatment, and for whom we were able to assign atleast two provider choices, one of them being the local PHC, and who had no missing information on key variables (Table 1). Outliers for OOP payment values were identified using the median absolute deviation (MAD) method; 12 outlier observations were capped at the threshold of Rs. 14,001. In the full sample, 3.8% of patients used the local PHC (including interactions with community health workers and lower public health facilities) and the proportion of patients using the district hospital was remarkably low (1.3%). In the study sample, visits to PHCs and district hospitals were combined, because of the low proportion of patients using district hospitals, resulting in 6.1% of patients visiting a PHC. The majority of patients (87%) visited some type of private provider, the majority of them being informal providers. Around 7% of patients visited a private hospital. OOP payments were lowest for patients visiting the local PHC and highest for private hospitals. Under half (43%) the patients were male, the average age was 30 years, and 20% reported that their illness was severe.

For the inter-linked survey, in the sub-sample of 57 villages, 1151 patients who were ill in the month before the survey visited 262 first-contact primary care providers, not including pharmacies and drug shops (Table 1). Notably, the vast majority of the primary care providers servicing these communities were informal providers (75%), followed by private AYUSH providers, the local PHC, and private MBBS doctors. The vast majority of primary care providers were male. The 13 villages excluded from the analysis due to few providers traced were similar to the study sample of 57 villages in terms of the percentage of sick individuals seeking treatment; however, in the excluded villages a greater proportion of sick individuals visited the local PHC, household distance to the PHC was higher, and households had lower asset ownership scores (results not shown).

Provider clinical competence for managing cases of hypertension, diarrhea, and pneumonia was measured using clinical vignettes. PHC clinicians had significantly higher average competence scores (40 out of 100) than other (private) providers (private MBBS, private AYUSH, and informal providers). There were no significant differences in competence scores between private MBBS, AYUSH, and informal providers (Table 1). Overall, all primary care providers in the study sample, irrespective of formal training or if they worked in the private or public sector, had low clinical competence as evidenced by the highest average competence score of 41 on a scale ranging from 0 to 100 (Table 1).

### 3.1 Proximity to primary care providers and care seeking

**Fig. 1:**
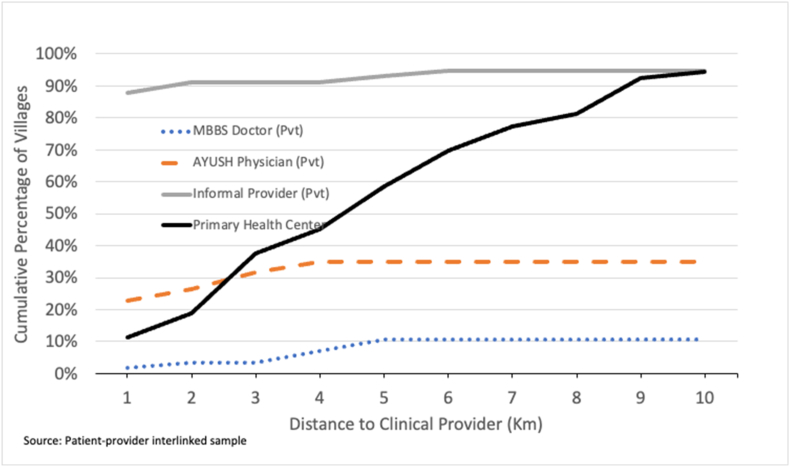
Village distance to primary care providers.

Most villages were in proximity to a primary care provider, though there was less geographic access to formally trained providers (Fig. 1). For example, around 88% of the villages had an informal provider within a distance of 1 km. On the other hand, only around 10% of villages had a PHC, 2% had an MBBS trained doctor (private) and 23% of the villages had an AYUSH physician (private) within a kilometer's distance. Within a distance of 5 km, which is about an hour's walk, about 60% of the villages had a PHC, 11% had an MBBS doctor (private), and 35% had AYUSH physician present. Nearly 40% of the villages did not have a qualified provider within a 5-km radius. Note PHCs can have either an MBBS doctor or AYUSH physician as the main clinician (Table 1). Notably, the proportion of villages within a kilometer of a PHC roughly doubles with each additional kilometer.

Within a 5-km radius of households in the study sample there were an average of 4.3 (95% CI 3.8, 4.8) primary care providers, of which, there was an average of 3.3 (95% CI 2.8, 3.8) informal providers, 0.6 (95% 0.4, 0.9) AYUSH practitioners, and 0.4 (95% CI 0.2, 0.5) MBBS doctors. In sum, there was no scarcity of primary care providers within walking distance of households, though there was a remarkable scarcity of trained medical doctors or AYUSH practitioners (results not shown).

**Fig. 2:**
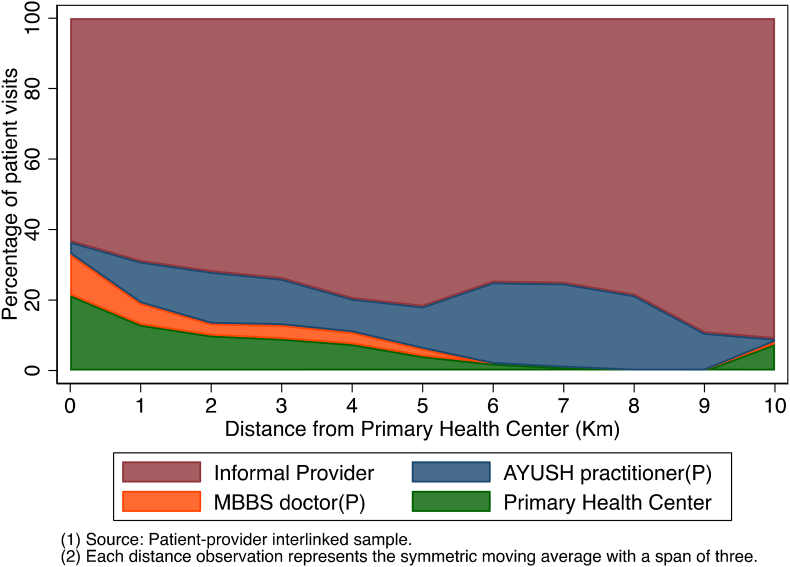
Percentage of patient visits by provider type and distance to PHC.

Fig. 2 shows how the percentage of patients visiting different types of primary care providers varies with distance of their residence to the PHC. Most patients visited an informal provider irrespective of proximity to the local PHC. Around 60% of the patients living in the vicinity (less than 1 Km) of a PHC, sought care from an informal provider. The probability of visiting an informal provider increased with distance to the local PHC. Only a minority (around 23%) of patients living in the vicinity of the PHC went there for treatment. Further, the proportion of patients visiting the PHC rapidly decayed as distance increased.

### 3.2 Quality of care

**Fig. 3:**
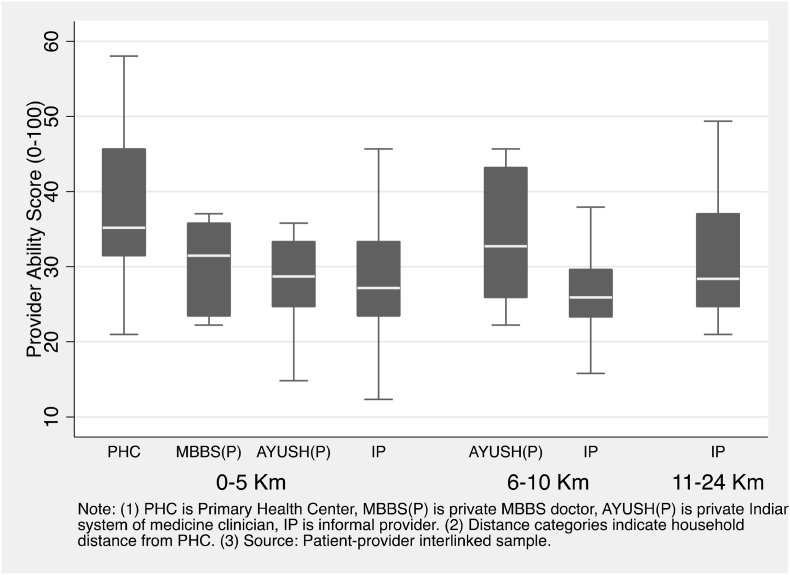
Competence of clinical providers seen by patients by household distance from Primary Health Center.

Of interest is to know if proximity to PHCs increased the likelihood of patients receiving better quality health care. Using clinical vignettes we measured clinical competence of primary care providers at PHCs in our study areas, as well as the primary care providers patients visited. Fig. 3 shows the clinical competence of clinicians at local PHCs, as well as private providers visited by patients seeking first contact care, by distance of the patient's residence from the local PHC. Clinicians at the local PHC (39.3) had the highest median competence. Private MBBS doctors (30.2), AYUSH practitioners (29.5), and informal providers (27.8) serving patients within 5 km of the local PHC, had similar median competence levels. Of note, there is considerable overlap in competence between provider types. For instance, half the PHCs had quality of care scores that were comparable to the majority of informal providers. More to the point, patients living close or far from the local PHC were likely to receive care from clinicians with similar competence irrespective of the type of provider they visited.

### 3.3 Choice of primary care provider

Of interest is to examine factors influencing choice of primary care providers. Table A1 in the appendix presents results from multinomial logistic regression (Model 1 and 2) and mixed multinomial logistic regression (Model 3 and 4). These models regress choice of provider on provider attributes (cost, distance, and clinical competence) and patient characteristics (age, sex, illness severity, wealth status). Models 2 and 4 include the correction for endogeneity using the control function approach; the cost residuals used in the cost function approach are statistically significant, suggesting the necessity for correcting for endogenous patient costs. Large changes in the regression coefficients related to patient costs are observed after controlling for endogeneity. We base our analysis on Model 4.

Patients choose between three types of providers – local PHC (includes government hospital), private clinic, and private hospital (Table A1). Increased distance between provider and patient residence significantly reduced the likelihood of the provider being selected (OR = 0.93 (0.91, 0.96)), implying that providers located farther away from the patient's residence were less likely to be visited. Higher OOP patient costs significantly lowered the likelihood of a provider being selected (OR = 0.36 (0.33, 0.39)); implying lower demand for more costly providers. Contrary to expectations, provider clinical competence had no significant effect on the likelihood of a provider being selected. The standard deviation (SD) estimates for the cost attribute indicate significant heterogeneity in the population with respect to the cost attribute; the effect of patient cost on the probability of selecting a provider has significant variation in the population. The estimated means and standard deviations of the regression coefficients provide information on the share of the population that places a positive or negative value on provider attributes (Table A1, Model 4). Approximately 77% of patients have a negative coefficient on the distance variable, indicating that distance had a negative inducement for these individuals. Almost all respondents had a negative coefficient on the cost variable, indicating that less costly providers was a positive inducement for individuals in the study sample.

The section “Patient attributes” (Table A1) indicates the association (relative risk ratio) between patient attributes and choice of private clinics or private hospitals, the reference category being PHCs. Males, older patients are significantly less likely to choose a private clinic or hospital over the local PHC. Patients with severe illness were more likely to select a private clinic over a PHC. Further, wealthier patients were less likely to select a private clinic over a PHC compared to poorer patients. For private hospitals, patients who were severely ill were 2.6 times more likely to visit a private hospital than a PHC. However, increase in household wealth was not associated with patients choosing private hospitals over PHC.

Table 2 presents marginal effects of key independent variables from the mixed multinomial logistic regression (Model 4). The figures in the table represent the probability of a particular provider (PHC, private clinic, private hospital) being selected due to a change in select provider attributes and patient characteristics. Reducing household distance to the PHC by 1 Km marginally increases the proportion of patients selecting PHCs by 0.04 percentage points, reduces the selection of private clinics by 0.04 percentage points and has no effect on the selection of private hospitals. If clinicians at all PHCs had the highest observed clinical competency score (76 out of 100), there would be no effect on the proportion of patients selecting PHCs or private clinics, or private hospitals. Reducing patient costs at PHCs by half would increase selection of PHCs by 1 percentage point, reduce the selection of private clinics by 1 percentage, and reduce by 0.1 percentage points patient selecting private hospitals.

**Table 2:** Marginal effects indicating the probability of patients selecting provider types due to changes in provider attributes and patient characteristics.

	PHC	Private Clinic	Private Hospital
Reduce household distance to PHC by 1 Km	0.0004 [0.0002, 0.0010]	−0.0004 [-0.0009, −0.0002]	−0.0001 [-0.0001, −0.00002]
Increase PHC clinician competence (76/100)	−0.0011 [-0.0039, 0.0018]	0.0010 [-0.0016, 0.0036]	0.0002 [-0.0025, 0.0005]
Reduce patient costs at PHC by 50%	0.0076 [0.0065, 0.0087]	−0.0070 [-0.0079, −0.0060]	−0.0009 [-0.0011, −0.0008]
Severe patient (ref: not severe)	−0.0019 [-0.0027, −0.0011]	−0.0326 [-0.0476, −0.0176]	0.0519 [0.0295, 0.0474]
Wealth quintile			
Quintile 2 (ref: Poorest 20%)	0.0029 [0.0019, 0.0038]	−0.0329 [-0.0485,- 0.0172]	0.0451 [0.0217, 0.0686]
Quintile 3 (ref: Quintile 2)	0.0012 [-0.0001, 0.0026]	−0.0267 [-0.0457, −0.0077]	0.0383 [0.0099, 0.0667]
Quintile 4 (ref: Quintile 3)	0.0050 [0.0037, 0.0062]	−0.0364 [-0.0529, −0.0199]	0.0472 [0.0225, 0.0719]
Richest 20% (ref: quintile 4)	0.0053 [0.0039, 0.0067]	−0.0563 [-0.0742, −0.0385]	0.0768 [0.0501, 0.1034]
Observations	19398	19398	19398

Note: Figures are marginal effects. This is the change in probability of a patient selecting a particular provider associated with a unit change in a continuous independent variable or a unit change from the reference group in a binary variable, with all other variables at their reference value. Multiplying these figures by 100 gives the percentage point change in a patient selecting a particular provider associated with a unit change in the independent variable. Marginal effects are based on regression results in Appendix A, Model 4.

Severely ill patients were less likely to visit PHCs by 0.2 percentage points, less likely to visit private providers by 3 percentage points, and more likely to visit private hospitals by 5 percentage points, compared to patients without severe illness. Wealthier individuals were marginally more likely to visit a PHC compared to those in the poorest wealth quintile. However, wealthier patients were less likely to visit a private clinic compared to those in the poorest quintile. For example, 6% fewer patients in the richest quintile selected a private clinic compared to the poorest wealth quintile. Greater wealth was associated with a higher probability of private hospitals being selected by patients. For example, 8 percentage points more patients in the richest quintile selected a private hospital compared to those in the poorest wealth quintile.

## 4 Discussion

Providing basic health services closer to where people live has been a longstanding policy objective in LMICs like India. Government efforts in expanding and resourcing networks of rural health centers is premised on the belief that without government intervention rural communities lack access to affordable quality primary care. Our study draws attention to the necessity for health policy to recognize the nature of rural health care markets so that the public sector presence in these markets can be more relevant to community health needs. The large-scale bypassing of PHCs observed in Bihar reflects its disconnect with local curative care needs and limits the agency of PHCs to influence the health of catchment populations. In general, across states of India, local PHCs are remarkably underutilized for curative care seeking. Making PHCs relevant to local health care needs requires a broader effort than simple actions such as increased geographical coverage, better infrastructure and staffing, or trainings to improve quality of clinical care. People need to see value in the services that PHCs offer for them to achieve their potential as a community health resource.

This study draws attention to several important features of rural health care markets in Bihar. First, primary care providers are abundant in rural Bihar; households were never far from a primary care provider, though they were from a qualified one. The majority of rural communities lived in proximity to a primary care provider; an average of 4.3 (95% CI 3.8, 4.8) providers were present within a 5-km radius of the households surveyed. There is, however, an acute scarcity of qualified primary care providers – only 40% of the surveyed villages were within 5 km of an MBBS doctor and 60% had a PHC within this radius. That 60% of households were within walking distance of a PHC is testimony to the reasonable success achieved by the public sector in providing health services close to communities, though issues such as absenteeism and resource constraints reduce this potential access. Informal providers, however, were the main first-contact primary care providers in rural Bihar. The ubiquity of informal providers in health care markets has been noted in several countries (Sudhinaraset et al., 2013). Second, PHCs were mostly bypassed^2^ by patients; even patients living close to a PHC sought care elsewhere. As such, geographical proximity of PHCs made only a marginal difference to the demand for their services. Third, the average quality of clinical care available at PHCs was better than other providers, but not by much. Half the PHCs possessed quality of care scores that were comparable to the majority of informal providers. As such, proximity to a PHC did not necessarily expose residents to better quality of care. Fourth, reducing distance to PHCs, improving quality of clinical care, or substantially reducing patient costs of seeking care at PHCs had only modest effects on the demand for their services.

The above findings have the following implications. That PHCs are largely bypassed by patients seeking curative services is an indication of their limited relevance to the curative care needs of their communities. Further, the scale at which PHCs are bypassed suggests that they have limited agency in influencing population health through facility-based curative services. Moreover, the potential financial protection offered by low-cost health services at PHCs is not achieved when patients bypass PHCs. Several studies, including this one, have documented the substantially lower OOP payments incurred by patients using PHCs compared to private care (Rao and Sheffel, 2018). Second, expanding the network of PHCs to bring services closer to communities, or providing training to improve clinician skills or reducing costs of health care at PHCs (e.g. by ensuring subsidized drugs are available) will likely only have marginal effects on the demand for curative services. Third, institutional engagement with private primary care providers is necessary, given their dominance in health care markets. The study findings are a call to re-think the organization of the public primary health care system, strategies to enhance relevance of PHCs to communities, and to develop policies that recognize and engage with pluralistic health care markets (Ahmed et al., 2013).

### 4.1 Bypassing primary health centers

Patients choose health care providers based on provider characteristics, as well, as their own circumstances such as illness severity, economic situation, and other characteristics. Various factors influence bypassing of health facilities. Well-known determinants include distance to the health facility, cost, and poor structural quality such as a lack of essential equipment, medicines, or diagnostics (Akin and Hutchinson, 1999; Bezu et al., 2021; Li et al., 2021). Patients are more likely to visit health facilities with more competent clinicians and where good prescription practices are followed (Leonard et al., 2002; Leonard, 2014). Patient factors associated with a higher likelihood of bypassing include being economically better-off, perceived poor quality of health services, duration of symptoms, and disease severity (Kahabuka et al., 2011; Kant et al., 2016; Khan et al., 2014; Karkee et al., 2015; Shah, 2016). Our analysis of provider choice in rural Bihar confirm some of these findings.

The vast majority (95%) of first-contact primary care visits made by rural patients were to private providers. Further, the majority of patients who lived in close proximity to a PHC sought private care elsewhere. The limited use of PHCs for curative care needs by local populations appears to be a feature across states of India, though there is interesting variability across the country reflecting the strength of the public health system (Rao and Sheffel, 2018). According to state survey reports, for example, the proportion of households usually seeking care at PHCs is 2.6% in Bihar, 3% in Uttar Pradesh, 8% in Madhya Pradesh, 19% in Odisha, 21% in Assam and Gujarat, 23% in Rajasthan, 22% in Kerala, and 34% in Tamil Nadu. (International Institute for Population Sciences (IIPS)) While these statistics highlight underutilization of PHCs for curative care seeking, it does not always follow that PHCs are bypassed to seek care with private providers; in some states (e.g. Odisha) care is instead sought at higher level public sector facilities.

In examining provider choice in rural Bihar, we find that patients might substitute between PHCs and private clinics when illness is not severe. This finding is perhaps not that puzzling. Though PHCs achieved the highest median quality of care among all primary care providers in Bihar, the overall levels were quite low indicating considerable scope for improving quality of care. Further, half the PHCs surveyed offered clinical quality of care that was comparable to that of the majority of informal providers. The quality of care offered at PHCs is also compromised by issues of absenteeism and structural quality; community reports in our study indicate that only 30% of the clinicians at PHCs were regularly present. However, improving quality of care will not be transformative by itself. As a study from another state in India demonstrated, even government health centers possessing highly competent clinicians and good infrastructure don't attract most local patients (Rao and Sheffel, 2018).

The pre-occupation of government policy with increasing geographic coverage of health services by building more primary health care facilities will only have modest effects on care seeking if most patients bypass them. Bypassing limits the ability of government health centers to influence individual and population health through personal health services. As discussed earlier, personal health services offered in health centers can contribute to large improvements in population health outcomes (Black et al., 2016; Prabhakaran et al., 2017; Berman). With the majority of patients in local communities staying away from PHCs, the latter have little information on the health of local communities outside of any routine disease surveillance, which also is weak in these contexts. Not having a finger fully on the health pulse of local communities is a major impediment to addressing their health needs (Black et al., 2016; Prabhakaran et al., 2017; Berman).

### 4.2 The challenge of making PHCs relevant to their communities

As we report in this study, increasing geographic proximity to health facilities, or improving clinician competence had only modest effects on demand for services. As such, building facilities close to communities, interventions to increase the clinical quality of care (e.g. by training), while important, is unlikely by themselves to transform PHCs in Bihar into the principal source of primary care for rural communities. As other studies indicate, in a pluralistic health care market, even well-resourced PHCs possessing a competent clinician who is regularly present, will be bypassed by the majority of patients seeking treatment irrespective of distance (Rao and Sheffel, 2018).

One approach for PHCs to gain relevance to local communities is to also focus on delivering Essential Public Health Functions (EPHF). Operationalization of EPHFs relies upon optimizing the role of PHCs in providing public health services, such as, community-based health promotion through education and counselling, disease screening, monitoring and evaluating population health status, health service utilization and surveillance of disease risk factors, immunization, and health outreach to increase access to care (WHO, 2021). This will necessitate a shift from a traditional disease‐focused approach towards one that gives importance to patient and community felt-needs in all aspects of service delivery. Strategies such as empanelment of the catchment population, regular home visits by community health workers targeted at neediest households, and team-based care can help health centers become more in tune with felt needs of the community.

Transforming PHCs so that they are relevant to the health care needs of their communities will require communities to see value in them; this requires a strong emphasis on community engagement to align with people's felt health needs. India's flagship HWC program has several of these features – population empanelment, team-based care, regular home visits; these need to become the central focus of the HWC program. In addition, embedding clinical care providers in the communities is also important. In this regard, contexts like Bihar will do well to look away from depending only on medical doctors to provide clinical services to rural populations; rather, dedicated cadres of rural non-physician clinicians, such as the recently introduced Community Health Officers in the HWC program, can become the embedded health resource for the communities they serve (Rao et al., 2013). Further, recruiting clinicians from within local communities, like some countries have done, can also support clinician-community integration (Pagaiya et al., 2015).

To better integrate with local communities, PHCs need to engage with other private primary care providers operating in their communities, including, informal providers, and local pharmacies/drug shops. The ubiquity of private providers in rural health care markets makes them important for efforts to improve health and service quality of rural communities (Gautham et al., 2014). Engagement with private providers can take several forms ranging from convening regular meetings to discuss local health issues, providing guidance on referrals, accreditation, and providing trainings. Such local networks of primary care providers led by the local government health center will enable the latter to better integrate with the communities they serve, be better informed about local health problems, and have greater agency in the health of the local population. Instances of formal engagement of informal providers are scarce globally and in India, though there have been several experiments with training informal providers. TB control programs have formally engaged with informal providers for detection, diagnosis, treatment and support with positive outcomes (Thapa et al., 2021). Similarly, efforts to engage community pharmacies have included clinical training of pharmacists/drug shops, policy and regulatory initiatives, and collaborative partnerships between public and private sector providers (Lamba et al., 2021.).

### 4.3 Limitations

This study has some notable limitations. Patient reports in the household survey of the type of provider they sought treatment from can be misleading in some instances. For example, patients may not distinguish between a Block PHC, a sub-district hospital, or a district hospital – all these are ‘government hospitals’ in a sense. We tried to correct for this by asking follow-up questions on the exact type of government facility they visited. Similarly, distinguishing among private doctors, AYUSH practitioners, traditional healers, and informal providers based on self-reports may be inaccurate since many of these providers practice western medicine to various degrees. Such issues were partly addressed in the patient-provider linked dataset by physically verifying the type of provider. However, for the choice modelling we included all these providers under “private clinics”. Third, the linked patient-provider dataset represented 57 of the 70 villages we surveyed where we were able to trace at least 40% of the visits to a provider. In these 57 villages too we were able to, in the aggregate, map 60% of the patient visits to a provider. So the loss in sample at the village and provider levels can create selection bias in the study results if the villages and providers left out were systematically different from those included in the analysis though we have no reason to believe that to be the case. The main reasons for excluding providers were if they were a drug shop or pharmacy, were not present when the study team visited, or were not traceable based on patient reports. Fourth, community health workers can play an important role in strengthening community ties to local PHCs and influence bypassing; we have not explored this in our study. Sixth, in analyzing provider choice we imputed provider characteristics for the alternatives not selected based on community averages. Earlier studies also address this issue similarly. Further, we didn't observe clinician quality at private hospitals and assumed that it would be around the average of private MBBS doctors. Seventh, in the provider choice analysis we focused on modelling where patients seek care, but did not model the decision to seek care because we did not observe attributes related to the “no use” choice. The latter analysis would provide insight into the provider attributes and patient characteristics that drive decisions to seek care, which is not available in the present study. Finally, we note that the distance variable in our regressions can be endogenous due to non-random provider placement, which would bias the distance estimates. Health providers could have located themselves in areas that have high demand or high population density. Similarly, people could also have moved to areas where health providers were present. Other sources of endogeneity include omitted variables that may affect choice and distance to health facilities. To correct for potential endogeneity in facility distance, several studies have used instrumental variables based on distance to nearest school, trading center, road, number of qualified teachers in nearest school or number of students in nearest school (McGuire et al., 2021; Kumar et al., 2014). Our surveys did not collect information on such variables and therefore we are unable to account for potential endogeneity of the distance variable. An added issue is that our choice model includes distance to three types of providers (PHC, private clinic, private hospital) and multiple instrumental variables would be required.

## 5 Conclusion

Government efforts in establishing networks of rural health centers are premised on the belief that government intervention is necessary for communities to have access to affordable quality primary care. The large-scale bypassing of PHCs observed in Bihar reflects their limited relevance to local healthcare felt needs in a context where their presence is needed most. Making communities value their local PHC requires a community-centered approach to the organization and delivery of primary care services.

## Funding

Bill and Melinda Gates Foundation, Delhi. Grant # OPP119434.

## Figures and Tables

**Table A1:** Results (odds ratios) from multinomial logit (Model 1 & 2) and mixed multinomial logit (Model 3 & 4) regression for provider choice.

	International (1978)	Berman (2000)	Perry et al. (2014)	Governmet of India (2018)
**Provider attributes**				
Clinical competence (0–100)	1.00 [0.99,1.00]	1.00 [0.99,1.02]	1.00 [0.99,1.00]	1.01 [0.98,1.02]
Distance (Km)	0.99** [0.98,1.00]	0.99* [0.98,1.00]	0.97* [0.93,1.00]	0.93** [0.91,0.96]
Log cost (Rs.)	0.97 [0.90,1.05]	0.39** [0.37,0.42]	1.12** [1.01,1.24]	0.36** [0.33,0.39]
SD (Log cost)			1.47** [1.40,1.55]	1.03** [1.00,1.05]
SD (Distance)			1.05* [1.00,1.10]	1.10** [1.06,1.14]
Residual control function		2.39** [2.34,2.44]		2.56** [2.44,2.69]
**Patient attributes**				
Private clinic (Ref: Primary health center)				
Male (Ref: female)	0.77** [0.64,0.94]	0.87** [0.78,0.98]	0.75** [0.60,0.93]	0.87** [0.77,0.98]
Age (years)	0.99** [0.99,0.99]	0.99** [0.99, 1.00]	0.99** [0.99,0.99]	0.99** [0.99,1.00]
Illness severe (Ref: not severe)	0.80* [0.63,1.02]	1.31** [1.13,1.52]	0.83 [0.63,1.10]	1.38** [1.17,1.62]
Wealth quintile (Ref: Poorest 20%)				
Wealth quintile 2	1.10 [0.84,1.45]	0.58** [0.49,0.68]	1.07 [0.78,1.47]	0.54** [0.45,0.65]
Wealth quintile 3	1.35 [0.92,1.97]	0.74** [0.59,0.94]	1.36 [0.89,2.09]	0.75** [0.58,0.97]
Wealth quintile 4	0.86 [0.65,1.16]	0.36** [0.31,0.43]	0.80 [0.57,1.12]	0.37** [0.30,0.44]
Richest 20%	0.92 [0.68,1.24]	0.32** [0.27,0.39]	0.84 [0.59,1.19]	0.34** [0.27,0.42]
Private hospital (Ref: Primary Health Center)				
Male (Ref: female)	0.73** [0.56,0.95]	0.73** [0.59,0.90]	0.73** [0.53,0.99]	0.74** [0.58,0.95]
Age (years)	0.99** [0.99,1.00]	0.99** [0.99,0.99]	0.99* [0.99,1.00]	0.99** [0.98,0.99]
Illness severe (Ref: not severe)	1.81** [1.34,2.44]	2.20** [1.75,2.77]	2.28** [1.55,3.34]	2.62** [1.98,3.46]
Wealth quintile (Ref: Poorest 20%)				
Wealth quintile 2	1.73** [1.14,2.63]	1.04 [0.73,1.47]	1.83** [1.10,3.03]	1.16 [0.76,1.77]
Wealth quintile 3	2.29** [1.36,3.86]	1.25 [0.82,1.91]	2.58** [1.38,4.84]	1.46 [0.88,2.42]
Wealth quintile 4	1.63** [1.06,2.51]	0.66** [0.46,0.94]	1.69* [0.99,2.91]	0.81 [0.52,1.25]
Richest 20%	2.32** [1.51,3.57]	0.82 [0.57,1.18]	2.54** [1.47,4.38]	1.06 [0.68,1.64]
N (observations)	19398	19398	19398	19398
N (patients)	7279	7279	7279	7279
Log likelihood	−3128	−1696	−3083	−1689

Note (International, 1978): Figures in parenthesis are 95% CI (Berman, 2000); *p < 0.10, **p < 0.05 (Perry et al., 2014); Model 1 and 3 have endogenous cost and models 2 and 4 use the control function for addressing endogeneity in costs (Governmet of India, 2018); Residual control function is the residual of the cost control function (GOI. Ayushman Bharat Health, 2022); Observations are clustered at the individual level.

## Data Availability

Data will be made available on request.

## Footnotes

1 Physicians trained in Indian systems of medicine (Ayurveda, Yoga, Unani, Siddha, Sowa-Rigpa, Homeopathy, Naturopathy), collectively known by the acronym AYUSH, serve at Primary Health Centers in many states of India.

2 Here we consider a PHC bypassed when a patient chooses not to visit their local PHC and seeks care at other primary care providers. By design, PHCs are expected to serve all the common healthcare needs of their local catchment population.
